# Supplementary material for: Predictive learning shapes the representational geometry of the human brain
Source: Nat Commun. 2024 Nov 8;15:9670. doi: 10.1038/s41467-024-54032-4 (PMC11549346; doi:10.1038/s41467-024-54032-4)
Supplement: Supplementary file 1 — Supplementary Information [file 41467_2024_54032_MOESM1_ESM.pdf]

Supplementary Material

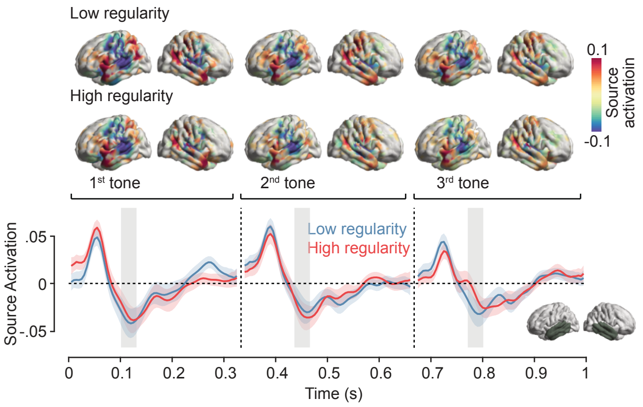

Fig. S1 I Stimulus space and auditory cortical responses. Top: Cortical distribution of evoked activity 100-120 ms post onset of each of the three tones in a triplet. Bottom: source-reconstructed evoked activity in bilateral temporal cortices (bottom right inset) across triplets in the low and high regularity sequences. Shaded areas indicate standard error of the mean (SEM).
