# Supplementary material for: Predictive learning shapes the representational geometry of the human brain
Source: Nat Commun. 2024 Nov 8;15:9670. doi: 10.1038/s41467-024-54032-4 (PMC11549346; doi:10.1038/s41467-024-54032-4)
Supplement: Supplementary file 2 — Reporting Summary [file 41467_2024_54032_MOESM2_ESM.pdf]

nature portfolio

Corresponding author(s): Antonino Greco, Markus Siegel

Last updated by author(s): May 6, 2024

Reporting Summary

Nature Portfolio wishes to improve the reproducibility of the work that we publish. This form provides structure for consistency and transparency in reporting. For further information on Nature Portfolio policies, see our Editorial Policies and the Editorial Policy Checklist.

Statistics

For all statistical analyses, confirm that the following items are present in the figure legend, table legend, main text, or Methods section.

n/a	Confirmed	
☐	☒	The exact sample size (*n*) for each experimental group/condition, given as a discrete number and unit of measurement
☐	☒	A statement on whether measurements were taken from distinct samples or whether the same sample was measured repeatedly
☐	☒	The statistical test(s) used AND whether they are one- or two-sided<br>*Only common tests should be described solely by name; describe more complex techniques in the Methods section.*
☐	☒	A description of all covariates tested
☐	☒	A description of any assumptions or corrections, such as tests of normality and adjustment for multiple comparisons
☐	☒	A full description of the statistical parameters including central tendency (e.g. means) or other basic estimates (e.g. regression coefficient) AND variation (e.g. standard deviation) or associated estimates of uncertainty (e.g. confidence intervals)
☐	☒	For null hypothesis testing, the test statistic (e.g. *F*, *t*, *r*) with confidence intervals, effect sizes, degrees of freedom and *P* value noted<br>*Give P values as exact values whenever suitable.*
☒	☐	For Bayesian analysis, information on the choice of priors and Markov chain Monte Carlo settings
☒	☐	For hierarchical and complex designs, identification of the appropriate level for tests and full reporting of outcomes
☐	☒	Estimates of effect sizes (e.g. Cohen's *d*, Pearson's *r*), indicating how they were calculated

Our web collection on statistics for biologists contains articles on many of the points above.

Software and code

Policy information about availability of computer code

Data collection	MEG data were collected using the acquisition software developed by the system manufacturer (CTF Systems Inc.)
Data analysis	All data analyses were carried out using MATLAB 2022a, the FieldTrip library (version 20220827) and the GCMI package (https://github.com/robince/gcmi)

For manuscripts utilizing custom algorithms or software that are central to the research but not yet described in published literature, software must be made available to editors and reviewers. We strongly encourage code deposition in a community repository (e.g. GitHub). See the Nature Portfolio guidelines for submitting code & software for further information.

Data

Policy information about availability of data

All manuscripts must include a data availability statement. This statement should provide the following information, where applicable:

- Accession codes, unique identifiers, or web links for publicly available datasets
- A description of any restrictions on data availability
- For clinical datasets or third party data, please ensure that the statement adheres to our policy

Source data are provided with this paper. Raw MEG data to reproduce all the results in our study are openly available at Zenodo via the following link: http://doi.org/10.5281/zenodo.3961467

Research involving human participants, their data, or biological material

Policy information about studies with human participants or human data. See also policy information about sex, gender (identity/presentation), and sexual orientation and race, ethnicity and racism.

Reporting on sex and gender	Participants were 12 females and 12 males
Reporting on race, ethnicity, or other socially relevant groupings	We randomized the sampling procedure for the ethnicity factor as possible given our local community ethnic distribution.
Population characteristics	Participants were 24 healthy volunteers (12 male) between 20 and 37 years old (mean age 27.54 years, SD = 9.96). All participants were right handed and had normal hearing abilities.
Recruitment	Participants were recruited via mailing lists at the University of Tübingen, Germany, as well as from the local community. Some participants were familiar with neuroscience or psychology in general, but not with the specific hypotheses of the study.
Ethics oversight	The study was approved by the local ethics committee of the Medical Faculty of the University of Tübingen (No. 231/2018BO1).

Note that full information on the approval of the study protocol must also be provided in the manuscript.

Field-specific reporting

Please select the one below that is the best fit for your research. If you are not sure, read the appropriate sections before making your selection.

[x] Life sciences   [ ] Behavioural & social sciences   [ ] Ecological, evolutionary & environmental sciences

For a reference copy of the document with all sections, see nature.com/documents/nr-reporting-summary-flat.pdf

Life sciences study design

All studies must disclose on these points even when the disclosure is negative.

Sample size	The sample size calculation was not based on analytical procedures but on the average sample size of the previous studies in this field.
Data exclusions	We did not exclude any participant from the data analyses.
Replication	We did not attempt to replicate our findings, as the main result was highly statistically robust.
Randomization	We did not use any randomization as there were no experimental groups.
Blinding	Blinding was not relevant, as there were no experimental groups.

Reporting for specific materials, systems and methods

We require information from authors about some types of materials, experimental systems and methods used in many studies. Here, indicate whether each material, system or method listed is relevant to your study. If you are not sure if a list item applies to your research, read the appropriate section before selecting a response.

Materials & experimental systems

n/a	Involved in the study	
[x]	[ ]	Antibodies
[x]	[ ]	Eukaryotic cell lines
[x]	[ ]	Palaeontology and archaeology
[x]	[ ]	Animals and other organisms
[x]	[ ]	Clinical data
[x]	[ ]	Dual use research of concern
[x]	[ ]	Plants

Methods

n/a	Involved in the study	
[x]	[ ]	ChIP-seq
[x]	[ ]	Flow cytometry
[x]	[ ]	MRI-based neuroimaging

Plants

Seed stocks	N/A
Novel plant genotypes	N/A
Authentication	N/A
